# Bicruciate Ligament Reconstruction in a Professional Rugby Player: Clinical Presentation and Literature Review

**DOI:** 10.1155/2015/962540

**Published:** 2015-09-29

**Authors:** Yoann Bohu, Shahnaz Klouche, Serge Herman, Antoine Gerometta, Nicolas Lefevre

**Affiliations:** ^1^Clinique du Sport Paris V, 36 boulevard Saint Marcel, 75005 Paris, France; ^2^Institut de l'Appareil Locomoteur Nollet, 23 rue Brochant, 75017 Paris, France; ^3^Racing-Metro 92, 11 avenue du Plessis, 92350 Le Plessis-Robinson, France

## Abstract

The association of an anterior cruciate ligament (ACL) tear and a posterior cruciate ligament (PCL) injury is rare in athletes, and to our knowledge it has never been described in a professional rugby player. We report the case of a 27-year-old international professional rugby player who presented with an ACL tear associated with chronic posterior laxity on a former PCL tear. The procedure associated arthroscopic ACL and PCL reconstruction in a one-stage operation with two autografts, bone-patellar tendon-bone and hamstring tendon, respectively. At 7 months postoperatively, the patient had returned to playing rugby at the same level of play. The therapeutic strategy successfully met the established goals of returning to sports at the same level of play with excellent functional results after 2 years of follow-up. A literature review was performed via PubMed. The inclusion criteria were the studies in English language, assessing the return-to-sport after bicruciate ligament reconstruction in athletes. Eight studies were included in analysis. Only one study has focused on the return-to-sport in 24 competitive athletes and two other studies have included 1 professional athlete each. The overall rate of the return-to-sport after bicruciate reconstruction varied between 100% and 50%.

## 1. Introduction

Anterior (ACL) and posterior (PCL) cruciate ligament tears are frequent and severe in professional rugby players [1, 2]. To our knowledge there is no published report of a recent ACL tear on a knee with a prior PCL tear in this population. The goal of treatment is to return rapidly to sports at the same level of play. An ACL tear in an athlete is an indication for ligament reconstruction, while, on the other hand, an isolated PCL tear with low grade laxity, with no associated injury of peripheral structures, is treated conservatively with functional rehabilitation for a rapid return-to-sports [3]. In the present case, a bicruciate ligament reconstruction was performed in a one-stage procedure.

## 2. Case Report

A 27-year-old patient who was an international professional rugby player consulted for a knee sprain that occurred during training after landing from a jump without player contact. He had a history of a sports injury in the same knee 8 years before during a rugby match. Since then he occasionally experienced patellofemoral pain syndrome during periods of rest from sports or when he stopped performing quadriceps strengthening exercises. The contralateral knee had been treated by arthroscopic meniscectomy for a meniscal lesion.

The patient measured 1 m91 and weighed 105 kg with a varus morphotype. The clinical examination showed a positive Lachman test with a soft end point (++), a clearly positive pivot shift test in internal valgus rotation, a posterior drawer test at 70° and 90° with a lack of end feel, spontaneous disappearance of the anterior tibial tubercle in the resting position, and tibial retraction during isometric contraction of the hamstrings at 90°, with no other anomalies.

**Figure 1:**
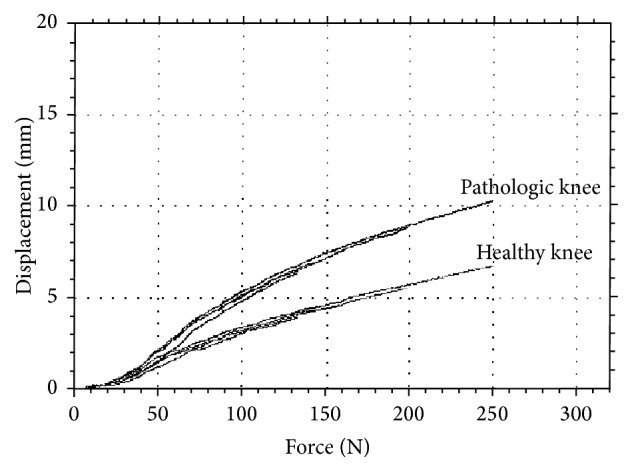
Preoperative anterior laximetry: graph of GNRB measurements.

MRI showed a recent full-thickness ACL tear, a lateral meniscal lesion, and an abnormal signal of the PCL. Laximetry with the GNRB (GeNouRoB) arthrometer [4] showed an anterior laxity differential of 2.5 and 3.7 mm for loads of 134 and 250 N, respectively (Figure 1).

Telos stress radiography performed with the knee in 90° flexion with and without hamstring contraction showed maximum posterior laxity of 13 mm in the right knee with a differential of 8.6 mm compared to the left knee (Table 1).

**Figure 2:**
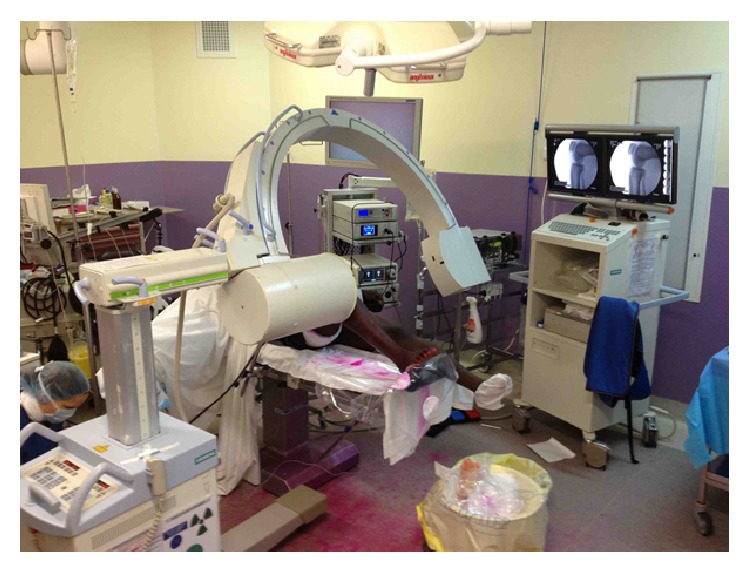
Installation of patient for fluoroscopy guided bicruciate reconstruction.

Following a preoperative physical reeducation protocol, the procedure was performed 23 days after the injury on a dry, mobile, and pain-free knee. The procedure was performed in a single-stage operation (Figure 2).

**Figure 3:**
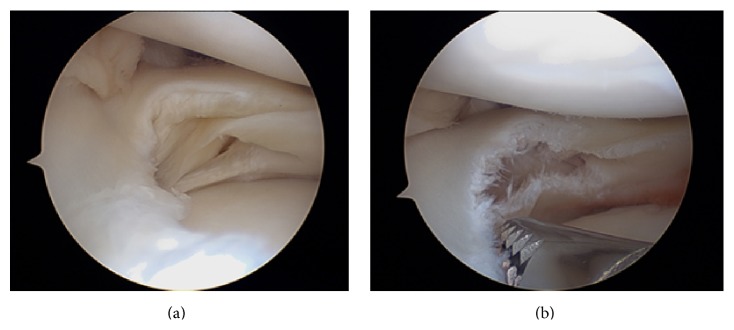
(a) Lateral meniscus complex lesion. (b) Lateral meniscus after minimal resection.

The semitendinosus and gracilis (STG) tendons were harvested from a single vertical parapatellar portal and prepared as a single bundle with four strands. The middle part of the patellar ligament was harvested for the bone-patellar tendon-bone (BTB) graft. Arthroscopic evaluation showed ICRS (International Cartilage Repair Society) stage 2 chondropathies on the lateral facet of the patella and the lateral and medial condyles. The lateral meniscus presented with a complex tear including a radial tear at the junction of the middle posterior segment, freeing a displaced 7 mm fragment into the joint space and a horizontal cleavage tear separating the meniscus in two to the popliteal hiatus. A resection of the minimum necessary amount of meniscal tissue was performed (Figure 3).

**Figure 4:**
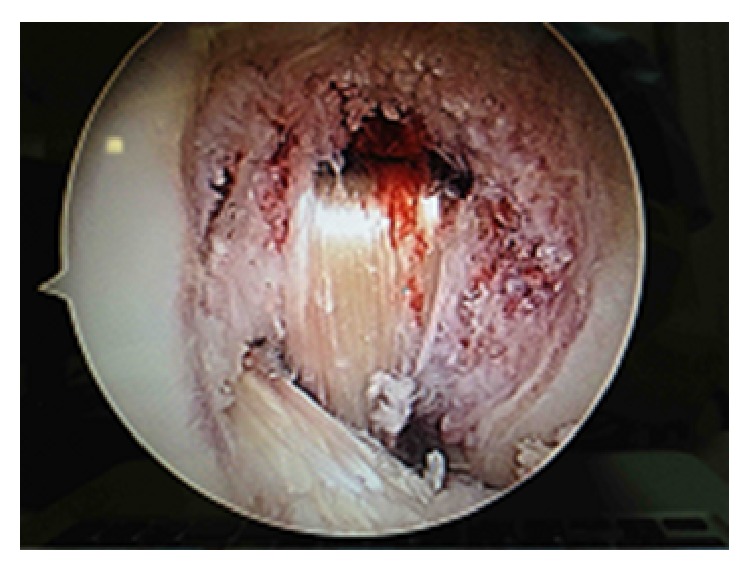
Perioperative view of the intracondylar notch after bicruciate reconstruction.

The blind femoral tunnel for BTB reconstruction was drilled in the lateral condyle through an anteromedial portal. The femoral tunnel for STG reconstruction of the LCP was drilled in the medial condyle by outside-in drilling. The tibial tunnel for STG reconstruction of the PCL was performed under fluoroscopic control through an accessory posteromedial portal, and then the tibial tunnel for BTB reconstruction of the ACL was drilled. After distal to proximal insertion of the STG graft, femoral fixation was obtained using an interference screw (Biosure, Smith & Nephew, USA) and then at 70° flexion by reducing posterior drawer under fluoroscopic control, tibial fixation was obtained with an interference screw and a ligament staple (Orthomed SAS, France). The BTB transplant was then inserted distally and attached in the femoral (SoftSilk, Smith & Nephew, USA) and tibial (Biosure, Smith & Nephew, USA) tunnels with interference screws (Figure 4). Immediate testing showed controlled anterior translation at 20° and posterior laxity at 90° flexion.

Postoperatively, the patient had an articulated brace and the knee was immediately free from 0 to 80° with full weight bearing. No PCL brace was used to control posterior laxity. The postoperative outcome was uneventful. The postoperative protocol was the same as ACL-type rehabilitation with closed kinetic chain exercises maintained for 5 full months. Protection of posterior drawer was not proposed, as in isolated PCL reconstruction.

Recovery was satisfactory at postoperative month 5. Isokinetic assessment of muscles was excellent. Concentric isokinetic knee extension tests at 90 and 240°/second and eccentric flexion isokinetic exercises at 30°/second were symmetric for both knees [13]. The proprioceptive single-hop and triple-hop tests were symmetric. The results of the objective and subjective IKDC [14], Lysholm [15], and ACL-RSI [16] scores were, respectively, Grade A, 83%, 100%, and 58.3%. On the other hand, the physical examination showed residual anterior laxity with a delayed hard end but no posterior drawer at 90°. The patient returned to professional competitive rugby 7 months after surgery. At postoperative month 12, the results of the objective and subjective IKDC, the Lysholm, and the ACL-RSI scores were, respectively, Grade A, 100%, 100%, and 95.8%. At 28 months of follow-up, the patient was still playing professional rugby.

## 3. Literature Review

**Figure 5:**
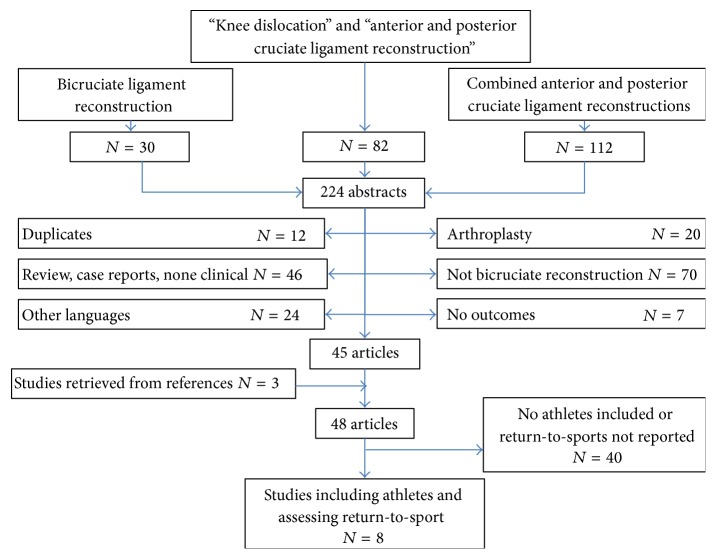
Flowchart of the systematic review.

An electronic search of the literature was performed in Medline via PubMed using the key words «bicruciate ligament reconstruction», «combined anterior and posterior cruciate ligament reconstructions», and «knee dislocation + anterior and posterior cruciate ligament reconstruction». The final search was performed on June 01, 2015. Moreover, the references were checked for each article and articles that could potentially be included in the analysis were manually searched for (Figure 5).

The main evaluation criterion was the return-to-sport (yes/no). Eight studies were included in analysis. Only one study has focused on the return-to-sport in 24 competitive athletes [11] and two other studies have included 1 professional athlete each [5, 10]. The overall rate of the return-to-sport varied between 100% [8] and 50% [10] (Table 2).

## 4. Discussion

This case study shows that ACL and PCL reconstruction in a single-stage operation with two autografts in an international professional rugby player makes possible a safe return-to-sports rapidly at the same level of play with excellent functional results after 2 years of follow-up.

In the present study several aspects were considered when determining the therapeutic strategy: whether to perform PCL reconstruction or not, in a one- or two-stage procedure, the type of reconstruction to be used, and the postoperative physical therapy protocol.

Certain arguments support functional physical therapy for the treatment of the PCL tear. Adding PCL reconstruction complicates the surgical procedure and can increase the risk of infection, which is already high in a professional athlete [17]. Finally, harvesting an additional graft also adds comorbidity. Functional treatment provides rapid and excellent short-term recovery and chronic posterior laxity is frequent and well tolerated in professional rugby players. The decision to perform bicruciate ligament reconstruction in a one-stage procedure was supported by several arguments. First, the prognosis for bicruciate ligament reconstruction is good in a professional player. Hirschmann et al. [11] reported results in a series of 26 elite players who underwent open surgery for bicruciate ligament tears associated with at least one lesion in a lateral ligament. Nineteen of the 24 patients evaluated after a mean of 8 years returned to sports 5.5 months after surgery. Also, failure to identify posterior laxity or the presence of posterior subluxation is one of the causes of failure of isolated ACL reconstruction. Weiler et al. [18] reported a series of 180 cases of isolated PCL reconstructions including 20 that had already been operated on for ACL reconstruction. In 4 cases the isolated ACL reconstruction resulted in posterior subluxation of the tibia, which required secondary bicruciate ligament reconstruction. Finally even in case of a partial PCL tear, cases of posterior instability persist following isolated ACL reconstruction [19].

The second therapeutic decision to be made was the type of reconstruction. For the ACL the autograft can be either the patellar tendon if the STG has already been harvested, the fascia lata [20], or the quadricipital tendon. If the quadricipital tendon is harvested, the second reconstruction can be performed using the STG or fascia lata, because harvesting of the patellar tendon can jeopardize rehabilitation of the extensor apparatus. The choice was made to use the STG graft for the PCL and the BTB for the ACL, both of which were harvested from a single anterointernal portal. The BTB was chosen instead of the fascia lata to obtain an intra-articular graft that was large enough to be used in a patient weighing 105 kg and to avoid additional comorbidities associated with an external approach. For the same reason we did not perform any extra-articular anterolateral articular tenodesis which we usually perform in professional players [21].

The third therapeutic decision was the choice of physical rehabilitation protocol. Because the goal was rapid recovery, we chose a rehabilitation protocol without posterior drawer protection, without restriction on the rehabilitation program or the use of a PCL brace. This compromise favors recovery of joint range of motion and muscular strength at the expense of residual posterior laxity. The articulated brace was immediately free from 0 to 80° with full weight bearing.

Although the clinical and functional results were satisfactory, persistent laxity remained. Extra-articular tenodesis would have been useful.

## 5. Conclusion

A bicruciate ligament tear in a professional athlete practicing a pivot sport is not frequent. There is no consensus on the therapeutic strategy in this case. The therapeutic decisions that were made resulted in an uneventful postoperative outcome, return-to-sports at a professional level after a short recovery period, and excellent functional results more than 2 years after surgery. The systematic review has shown a lack of data for professional athletes.

## Figures and Tables

**Table 1:** Preoperative posterior laximetry on X-rays at 90° flexion with and without hamstring contraction.

	Right (injured)	Left (healthy)
**At rest**	7.5 mm	3 mm
**Hamstring contraction**	13 mm	4.4 mm
Differential rest/contraction	5.5 mm	1.4 mm
Differential right/left knee	**(+) 8.6 mm right**	

**Table 2:** Review of the literature.

Authors	Year	Athletes	Age (years)	Sex	Sport	Level of sport	Surgical technique	Follow-up	Return-to-sport	Level of return-to-sport
Sisto and Warren [5]	1985	13/19	37.7	NR	Baseball 1 NR 12	Professional 1 Competitive 12	Open (one stage)	24 m	8/13 (61.5%)	Same 8/8

Noyes and Barber-Westin [6]	1997	8/11	17–42	10 M 1 F	Jumping 4 Running 2 Swimming 2	NR	Arthroscopy (one stage)	2.5–9 y	7/8 (87.5%)	Increased 1/7 Same 2/7 Decreased 4/7

Wascher et al. [7]	1999	11/13	27.5 (14–51)	NR	NR	NR	Open 11 Arthroscopy 2 (one stage)	38.4 m (24–54)	10/11 (90.9%)	Same 6/10 Decreased 4/10

Xie et al. [8]	2007	10/10	34 (25–51)	8 M 2 F	NR	NR	Arthroscopy (one stage)	18 m (12–30)	10/10 (100%)	Same 8/10 Decreased 2/10

Zhao et al. [9]	2008	2/21	27 (18–56)	15 M 6 F	NR	NR	Arthroscopy (one stage)	24 m	2/2 (100%)	Same 2/2

Shi et al. [10]	2008	4/15	24 (17–44)	11 M 4 F	Judo 1 Wrestling 1 NR 2	Professional 1 Collegiate 2 Recreational 1	Arthroscopy (one stage)	38 m (36–40)	2/4 (50%)	Same 2/2

Hirschmann et al. [11]	2010	24/24	24 (14–32)	24 M	Soccer 16 Skiing 4 Fencing 2 Basketball 1 Handball 1	Competitive (national/international) 24	Open (one stage)	8 y (1–23)	19/24 (79.2%)	Same 8/19

Cartwright-Terry et al. [12]	2014	22/25	34 (23–50)	22 M 3 F	NR	NR	Arthroscopy (one stage)	5 y	23/25 (92%)	NR

## References

[1] Brooks J. H. M., Fuller C. W., Kemp S. P. T., Reddin D. B. (2005). Epidemiology of injuries in English professional rugby union. Part 1 match injuries. *British Journal of Sports Medicine*.

[2] Brooks J. H. M., Fuller C. W., Kemp S. P. T., Reddin D. B. (2005). Epidemiology of injuries in English professional rugby union: part 2 training injuries. *British Journal of Sports Medicine*.

[3] LaPrade C. M., Civitarese D. M., Rasmussen M. T., LaPrade R. F. (2015). Emerging updates on the posterior cruciate ligament: a review of the current literature. *The American Journal of Sports Medicine*.

[4] Robert H., Nouveau S., Gageot S., Gagnière B. (2009). A new knee arthrometer, the GNRB: experience in ACL complete and partial tears. *Orthopaedics and Traumatology: Surgery and Research*.

[5] Sisto D. J., Warren R. F. (1985). Complete knee dislocation. A follow-up study of operative treatment. *Clinical Orthopaedics and Related Research*.

[6] Noyes F. R., Barber-Westin S. D. (1997). Reconstruction of the anterior and posterior cruciate ligaments after knee dislocation. Use of early protected postoperative motion to decrease arthrofibrosis. *The American Journal of Sports Medicine*.

[7] Wascher D. C., Becker J. R., Dexter J. G., Blevins F. T. (1999). Reconstruction of the anterior and posterior cruciate ligaments after knee dislocation. Results using fresh-frozen nonirradiated allografts. *The American Journal of Sports Medicine*.

[8] Xie F., Yang L., Guo L., Dai C., Han X.-S. (2007). A follow-up study of arthroscopic combined reconstruction of anterior and posterior cruciate ligaments with allograft patellar tendon. *Chinese Journal of Traumatology*.

[9] Zhao J., Huangfu X., He Y., Yang X., Zhu Y. (2008). Simultaneous double-bundle anterior cruciate ligament and posterior cruciate ligament reconstruction with autogenous hamstring tendons. *Arthroscopy*.

[10] Shi D.-H., Cai D.-Z., Wang K., Rong L.-M., Xu Y.-C. (2008). Concurrent arthroscopic bicruciate ligament reconstruction using Achilles tendon-bone allografts: Experience with 15 cases. *Chinese Journal of Traumatology—English Edition*.

[11] Hirschmann M. T., Iranpour F., Müller W., Friederich N. F. (2010). Surgical treatment of complex bicruciate knee ligament injuries in elite athletes: what long-term outcome can we expect?. *The American Journal of Sports Medicine*.

[12] Cartwright-Terry M., Yates J., Tan C. K., Pengas I. P., Banks J. V., McNicholas M. J. (2014). Medium-term (5-year) comparison of the functional outcomes of combined anterior cruciate ligament and posterolateral corner reconstruction compared with isolated anterior cruciate ligament reconstruction. *Arthroscopy*.

[13] Croisier J.-L., Forthomme B., Namurois M.-H., Vanderthommen M., Crielaard J.-M. (2002). Hamstring muscle strain recurrence and strength performance disorders. *American Journal of Sports Medicine*.

[14] Anderson A. F., Irrgang J. J., Kocher M. S., Mann B. J., Harrast J. J. (2006). The International Knee Documentation Committee Subjective Knee Evaluation Form: normative data. *The American Journal of Sports Medicine*.

[15] Lysholm J., Gillquist J. (1982). Evaluation of knee ligament surgery results with special emphasis on use of a scoring scale. *The American Journal of Sports Medicine*.

[16] Bohu Y., Klouche S., Lefevre N., Webster K., Herman S. (2015). Translation, cross-cultural adaptation and validation of the French version of the Anterior Cruciate Ligament-Return to Sport after Injury (ACL-RSI) scale. *Knee Surgery, Sports Traumatology, Arthroscopy*.

[17] Sonnery-Cottet B., Archbold P., Zayni R. (2011). Prevalence of septic arthritis after anterior cruciate ligament reconstruction among professional athletes. *The American Journal of Sports Medicine*.

[18] Weiler A., Jung T. M., Lubowicki A., Wagner M., Schöttle P. B. (2007). Management of posterior cruciate ligament reconstruction after previous isolated anterior cruciate ligament reconstruction. *Arthroscopy*.

[19] Wolf R. S., Lemak L. J. (2002). Incomplete bicruciate knee injuries: results of treatment with isolated anterior cruciate ligament reconstruction. *Arthroscopy*.

[20] Khiami F., Wajsfisz A., Meyer A., Rolland E., Catonné Y., Sariali E. (2013). Anterior cruciate ligament reconstruction with fascia lata using a minimally invasive arthroscopic harvesting technique. *Orthopaedics & Traumatology: Surgery & Research*.

[21] Christel P., Djian P. (2002). Anterio-lateral extra-articular tenodesis of the knee using a short strip of fascia lata. *Revue de Chirurgie Orthopédique et Réparatrice de l Appareil Moteur*.

